# The landscape and prognostic value of tumor-infiltrating immune cells in gastric cancer

**DOI:** 10.7717/peerj.7993

**Published:** 2019-12-10

**Authors:** Linhai Li, Yiming Ouyang, Wenrong Wang, Dezhi Hou, Yu Zhu

**Affiliations:** Department of General Surgery, the First People’s Hospital of Yunnan Province, the Affiliated Hospital of Kunming University of Science and Technology, Kunming, China

**Keywords:** Tumor-infiltrating immune cells, Nomogram, Gastric cancer

## Abstract

**Background:**

Gastric cancer (GC) is the fourth most frequently diagnosed malignancy and the second leading cause of cancer-associated mortality worldwide. The tumor microenvironment, especially tumor-infiltrating immune cells (TIICs), exhibits crucial roles both in promoting and inhibiting cancer growth. The aim of the present study was to evaluate the landscape of TIICs and develop a prognostic nomogram in GC.

**Materials and Methods:**

A gene expression profile obtained from a dataset from The Cancer Genome Atlas (TCGA) was used to quantify the proportion of 22 TIICs in GC by the CIBERSORT algorithm. LASSO regression analysis and multivariate Cox regression were applied to select the best survival-related TIICs and develop an immunoscore formula. Based on the immunoscore and clinical information, a prognostic nomogram was built, and the predictive accuracy of it was evaluated by the area under the curve (AUC) of the receiver operating characteristic curve (ROC) and the calibration plot. Furthermore, the nomogram was validated by data from the International Cancer Genome Consortium (ICGC) dataset.

**Results:**

In the GC samples, macrophages (25.3%), resting memory CD4 T cells (16.2%) and CD8 T cells (9.7%) were the most abundant among 22 TIICs. Seven TIICs were filtered out and used to develop an immunoscore formula. The AUC of the prognostic nomogram in the TCGA set was 0.772, similar to that in the ICGC set (0.730) and whole set (0.748), and significantly superior to that of TNM staging alone (0.591). The calibration plot demonstrated an outstanding consistency between the prediction and actual observation. Survival analysis revealed that patients with GC in the high-immunoscore group exhibited a poor clinical outcome. The result of multivariate analysis revealed that the immunoscore was an independent prognostic factor.

**Discussion:**

The immunoscore could be used to reinforce the clinical outcome prediction ability of the TNM staging system and provide a convenient tool for risk assessment and treatment selection for patients with GC.

## Introduction

Gastric cancer (GC) is the fourth most frequently diagnosed malignancy and the second leading cause of cancer-associated mortality worldwide (Sitarz et al., 2018; Wang et al., 2016). Annually, it is estimated that approximately one million new cases of GC are diagnosed and there are >800,000 cases of GC-associated mortality (Park et al., 2018). With the advancements in treatment and diagnostic technology, the clinical prognosis of patients with GC has been significantly improved (Wu et al., 2019). However, for patients in advanced stage the 5-year relative survival rate remains limited to only 20% (Wei et al., 2017). What’s worse is that, due to a lack of classical symptoms in the early stage, the majority of GC patients are in the advanced stage at the time of diagnosis and almost 50% of patients have experienced metastasis (Wang et al., 2018a; Wang et al., 2018b). TNM staging classified by the American Joint Committee on Cancer and International Union against Cancer and histological subtype are the most commonly used clinicopathological variables for clinical decision making and prognosis stratification of GC (Jiang et al., 2018). However, an increasing number of studies have reported differences in clinical outcomes among GC patients with the same TNM stage and similar therapeutic regimens (Noh et al., 2014; Zeng et al., 2018), suggesting that TNM staging alone cannot provide complete information for prognosis prediction of GC.

The tumor microenvironment (TME) is a complicated system consisting of extracellular matrix, chemokines, cytokines and non-tumor cells (Yang et al., 2018). Tumor-infiltrating immune cells (TIICs) are a component of non-tumor cells in TME. Several studies have reported crucial functions of TIICs both in promoting and inhibiting cancer growth as independent prognostic factors in various cancer types (Xiong et al., 2018; Liu et al., 2018). In addition, novel insights about the role of TIICs support that the composition of TIICs along with their functionality may be relevant for cancer management (Bense et al., 2016). However, conventional detection technology for TIICs, such as immunohistochemistry (IHC) and flow cytometry, are based on several marker proteins and are not capable of systematically evaluating the functions of diverse immune cells, due to the restriction of the number of markers that cannot be measured simultaneously with current methods (Zhou et al., 2019). As an alternative, the CIBERSORT algorithm can simultaneously determine the landscape of 22 TIICs by assessing the relative expression changes of a set of barcode genes (521 genes) compared with the expression of all other genes in the sample with a support vector regression approach (Newman et al., 2015). The CIBERSOFT algorithm has been considered to be the most accurate method for identifying TIICs and is used to develop the immunoscore model in several cancer types (Bense et al., 2016; Liu et al., 2017).

The present study applied the CIBERSORT algorithm for analysis of gene expression profiles from The Cancer Genome Atlas (TCGA; https://cancergenome.nih.gov) to quantify proportions of 22 TIICs in GC and develop an immunoscore model, which was validated by data from the International Cancer Genomics Consortium (ICGC) dataset. By integrating the immunoscore and clinical information, a prognostic nomogram for predicting the 3-year overall survival (OS) in GC was constructed. A novel prognostic nomogram is required for improvement of treatment selection and outcome prediction compared with TNM staging, and may assist with the development of novel strategies for diagnosis and the identification of potential drug targets of GC.

## Materials and Methods

### Data profile

The gene expression profiles were obtained from TCGA and ICGC datasets (downloaded in April 2019), and then subjected to background correction and normalization with Perl 5.0 (http://www.perl.org/). Meanwhile, relevant clinical characteristics of cancer cases were also collected. Patients with a follow-up time <30 days or a lack of pathological diagnosis were excluded from the study. The differences of demographic and baseline characteristics between two sets were compared by 2 tests, using SPSS 20.0 (IBM Corp.). *P* < 0.05 was considered to indicate a statistically significant difference.

### Evaluation of tumor-infiltrating immune cells

As described previously (Liu et al., 2017), the CIBERSORT method was used to quantify the proportions of 22 TIICs both in GC samples and normal samples using the LM22 signature and 1,000 permutations. Cases with CIBERSORT *P* < 0.05, which reflected that the deconvolution results were accurate, would be selected for further analysis. In the present study, a total of 222 samples (15 normal samples and 207 GC samples in TCGA dataset; 52 GC samples in the ICGC dataset) were filtered out. Group comparisons of 22 TIICs proportions were performed by *t*-test between normal and GC samples, and one-way ANOVA among different TNM stages using SPSS 20.0 (IBM Corp.). *P* < 0.05 was considered to indicate a statistically significant difference.

### Construction and evaluation of a nomogram

LASSO Cox regression analysis was used to select TIICs that were highly associated with the overall survival of GC patients among the 22 TIICs. Subsequently, candidate TIICs were subjected to multivariate Cox regression analysis to further screen out the best survival-related candidate TIICs and develop an immunoscore formula. On the basis of immunoscore, patients were divided into low- and high-immunoscore groups. Ultimately, a prognostic nomogram integrating immunoscore and clinical information was developed for predicting 3-year survival probabilities of GC patients. Meanwhile, a calibration plot with bootstrapping set to 1,000 resamples and receiver operating characteristic curve (ROC) analysis was performed to assess the predictive capacity of the prognostic nomogram by calculating the area under the curve (AUC). Similarly, ROC curve analyses of TNM stage alone, the ICGC set and the whole set were also performed to validate the prognostic nomogram.

### Expression analysis of differentially expressed genes (DEGs) between low- and high immunoscore groups

The statistical software program R (version 3.5.2; R Core Team, 2018) and the Bioconductor package edgeR (http://www.bioconductor.org/packages/release/bioc/html/edgeR.html) were used to identify DEGs between the low- and high-immunoscore groups, with the criteria of —log(fold-change)—>1.5 and false discovery rate <0.05.

### Functional enrichment analysis and protein-protein interaction (PPI) network construction

All DEGs between the low- and high-immunoscore groups were utilized for Kyoto Encyclopedia of Genes and Genomes (KEGG) and Gene Ontology (GO) analysis with adjusted *P* < 0.01 as the threshold. Meanwhile, all DEGs were inputted into STRING (https://string-db.org) (Szklarczyk et al., 2019) to predict protein-protein interactions, with a confidence >0.9 as the cut-off criterion. Data of the PPI network were processed by Cytoscape and genes in significant modules (MCODE score >10 and number of nodes >10) were extracted from the PPI network as the most important targets of differing immunoscores and were used for further analysis.

### Survival analysis

Kaplan–Meier analysis and a log-rank test were performed to construct a survival curve and assess the survival difference between the low- and high-immunoscore groups, and analyze the association of the proportions of 22 TIICs with overall survival. In addition, univariate and multivariate Cox regression models were used to determine independent prognostic factors. *P* < 0.05 was set as cut-off value.

### Expression profile of immunomodulators

Immune checkpoint inhibitors have revolutionized cancer therapy and have been approved for various cancer treatments. In the present study, several key immunomodulators (LAG3, TIM3, NKG2A, VISTA, CTLA-4, IFNG, IL2, IL6, ICOS, ICAM1, TIGIT, PD-1 and PD-L1) were quantified both in normal samples and GC samples. The differences in expression of the immunomodulators between normal and GC samples, as well as low- and high-immunoscore groups, were compared by *t*-test.

## Results

### Patient characteristics

After cases with a follow-up time <30 days and CIBERSORT *P* > 0.05 were removed, 274 samples were enrolled in the present study, including 15 normal gastric samples and 207 GC samples in TCGA set and 52 GC samples in the ICGC set. All 259 patients were diagnosed pathologically with GC. The detailed demographic and baseline characteristics of the 259 GC patients are presented in Table 1. There were no statistically significant differences in the clinical information, including age, sex, ethnicity, tumor stage, immunoscore and survival status, between the two datasets.

**Table 1:** Clinical factors of patients with CIBERSORT *p*-value <0.05.

Parameter	TCGA dataset (*n* = 207)	ICGC dataset (*n* = 52)	Whole set (*n* = 259)	*P* value
Age (years)				0.742
<60	70(33.8%)	16(30.8%)	86(33.2%)	
≥60	137(66.2)	36(69.2%)	173(66.8%)	
Gender				0.692
Male	128(61.8%)	33(63.5%)	161(62.2%)	
Female	79(38.2%)	19(35.5%)	98(37.8%)	
Race				0.872
White	137(66.2%)	33(63.5%)	170(65.6%)	
Black	13(6.3%)	4(7.7%)	17(6.6%)	
Asian	43(20.7%)	10(19.2%)	53(20.5%)	
Other	14(6.8%)	5(9.6%)	19(7.3%)	
Pathologic Stage				0.165
Stage i	28(13.5%)	8(15.4%)	36(13.9%)	
Stage ii	70(33.8%)	15(28.9%)	85(32.8%)	
Stage iii	85(41.1%)	19(36.5%)	104(40.2%)	
Stage iv	24(11.6%)	10(19.2%)	34(13.1%)	
Immunoscore				0.765
Low	104(50.2%)	26(50%)	130(50.2%)	
High	103(49.8%)	26(50%)	129(49.8%)	
Survival status				0.127
Alive	121(58.5%)	37(71.2%)	158(61.0%)	
Dead	86(41.5%)	15(28.8%)	101(39.0%)	

### Evaluation of tumor-infiltrating immune cells

**Figure 1:**
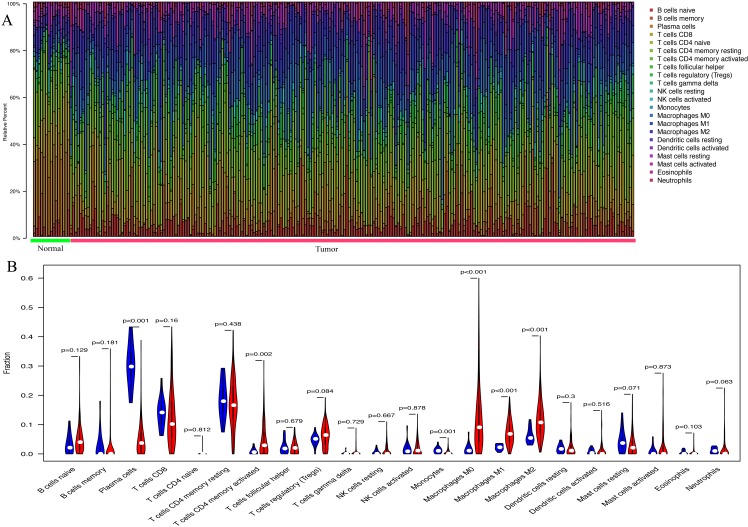
The landscape of 22 tumor-infiltrating immune cells. (A) The proportions of 22 TIICs in each samples quantified by CIBERSORT (B) The difference of the proportions of 22 TIICs between normal samples and GC sample.

With the CIBERSORT method, the present study quantified the proportions of 22 TIICs both in GC samples and normal samples, including naïve B cells, memory B cells, plasma cells, naïve CD4 T cells, resting memory CD4 T cells, activated memory CD4 T cells, CD8 T cells, gamma delta T cells, T follicular helper cells, regulatory T cells, resting dendritic cells, activated dendritic cells, macrophages (M0, M1 and M2), resting NK cells, activated NK cells, resting mast cells, activated mast cells, monocytes, neutrophils and eosinophils (Fig. 1A). In normal gastric samples, the proportion of plasma cells (29.3%) was the most abundant among 22 TIICs, followed by resting memory CD4 T cells (17.5%) and CD8 T cells (13.8%). However, in GC samples, the highest proportion among the 22 TIICs was macrophages (25.3%), followed by resting memory CD4 T cells (16.2%) and CD8 T cells (9.7%). As presented in Fig. 1B, the percentage of plasma cells (*P* < 0.001) and monocytes (*P* = 0.001) in GC samples was significantly lower than that in normal gastric samples, whereas, the fraction of activated memory CD4 T cells (*P* = 0.002) and macrophages (M0, *P* < 0.001; M1, *P* < 0.001; M2, *P* < 0.001) was opposite.

### Construction and evaluation of a prognostic nomogram

**Figure 2:**
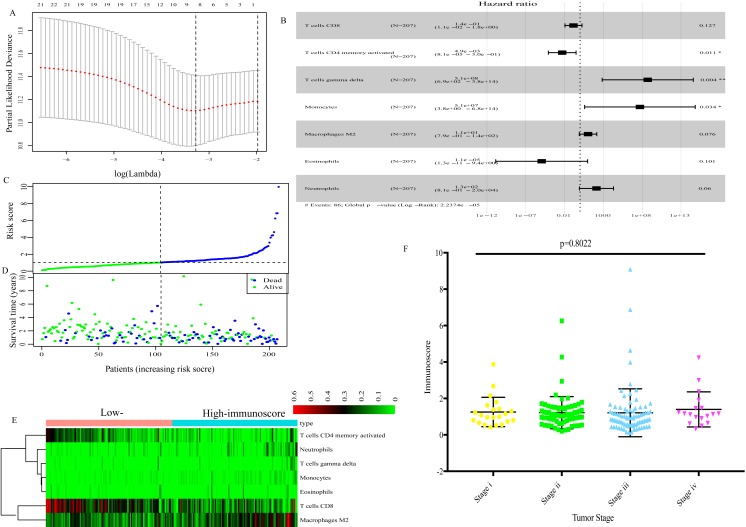
The immunoscore based on 7 TIICs (CD8 T cells, activated memory CD4 T cells, gamma delta T cells, monocytes, macrophages M2, neutrophils and eosinophils). (A) LASSO regression coefficient profiles of survival-associated TIICs. (B) The forest map of multivariate Cox regression analysis. (C), (D), (E) Distribution of immunoscore, survival statuse and expression profile of 7 TIICs of each patient. (F) Distribution of immunoscore among among different TNM staging.

Using the LASSO Cox regression model (Fig. 2A), TIICs that were highly correlated with the OS of GC patients were determined and subjected to multivariate Cox regression analysis to identify the best survival-related TIICs. Ultimately, seven TIICs (CD8 T cells, activated memory CD4 T cells, gamma delta T cells, monocytes, macrophages M2, neutrophils and eosinophils) were filtered out and used to develop an immunoscore formula (Fig. 2B). Based on the immunoscore, GC patients were divided into low- and high-immunoscore groups. The distribution of immunoscore, survival status and expression profile of the seven TIICs of each patient are presented in Figs. 2C–2E. No significant difference was identified between different TNM stages (Fig. 2F).

**Figure 3:**
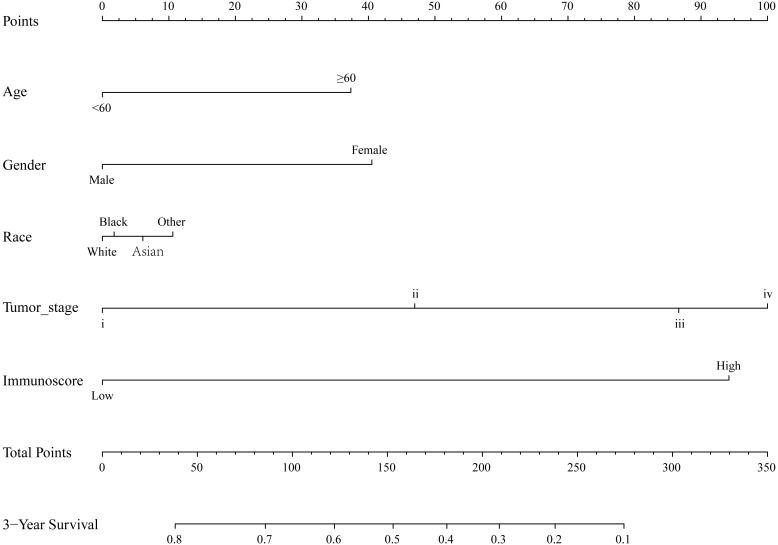
The prognostic nomogram for predicting 3-year survival probabilities of GC patients.

**Figure 4:**
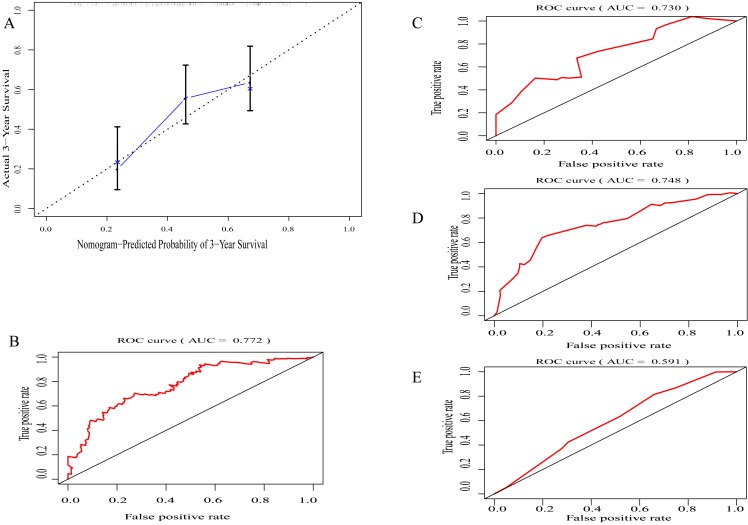
Evaluation of prognostic nomogram. (A) The calibration plot of prognostic nomogram for 3-year survival probability (B) The area under the receiver operating characteristic (ROC) curve (AUC) of prognostic nomogram in TCGA set, (C) in ICGC set (D) in whole set (E) TNM staging along.

By integrating clinical information and the immunoscore, a prognostic nomogram for predicting 3-year survival probabilities of GC patients was built (Fig. 3). The AUC of the prognostic nomogram and TNM stage alone in TCGA set was 0.772 and 0.591, respectively (Figs. 4B and 4E). In addition, the AUC of the ICGC set and whole set was 0.730 and 0.748, respectively (Figs. 4C and 4D). The calibration plot of the prognostic nomogram for 3-year survival probability is shown in Fig. 4A.

### Functional enrichment analysis and PPI network

**Figure 5:**
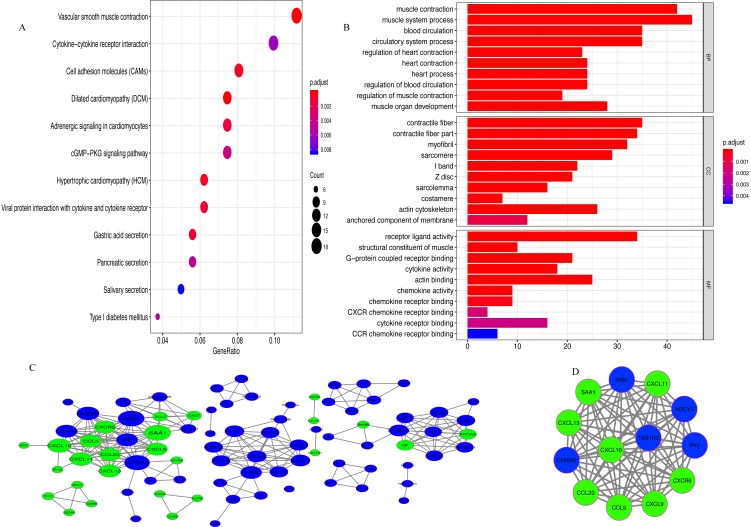
Analysis of DEGs. (A) The pathways enriched for 342 DEGs (B) GO enrichment analysis (C) Protein-protein interaction (PPI) network. Red and green nodes represent up-regulated and down-regulated genes, respectively (D) MCODE with scores >10 and number of nodes >10.

A total of 342 DEGs (257 upregulated and 85 downregulated genes) were determined between the low- and high-immunoscore groups (Fig. S1). The results of KEGG analysis revealed that 342 DEGs were predominantly involved in 13 pathways, among which, a few pathways were highly associated with the human immune system, such as ‘Cell adhesion molecules (CAMs)’, ‘Chemokine signaling pathway’ and ‘Cytokine-cytokine receptor interaction’ (Fig. 5A). In GO analysis (Fig. 5B; Table S1), 147 biological processes were related to 342 DEGs, including ‘T cell migration’ and ‘regulation of T cell migration’.

A PPI network of DEGs with confidence >0.9 was gathered from the online STRING database and then processed by Cytoscape. There were 86 nodes (60 upregulated and 26 downregulated genes) and 262 edges in the PPI network (Fig. 5C). With MCODE score >10 and number of nodes >10 as the cut-off values, a MCODE containing 13 genes (CCL20, CXCL10, SAA1, CCL5, CXCR6, CXCL13, CXCL11, CXCL9, ADCY5, PYY, TAS1R3, NMU and CHRM2) was extracted from the PPI network and used for further analysis (Fig. 5D). KEGG analysis revealed that 13 genes in MCODE were mainly enriched in the following four pathways: ‘Chemokine signaling pathway’, ‘Cytokine-cytokine receptor interaction’, ‘Toll-like receptor signaling pathway’ and ‘TNF signaling pathway’ (Fig. S2).

### Survival analysis

**Figure 6:**
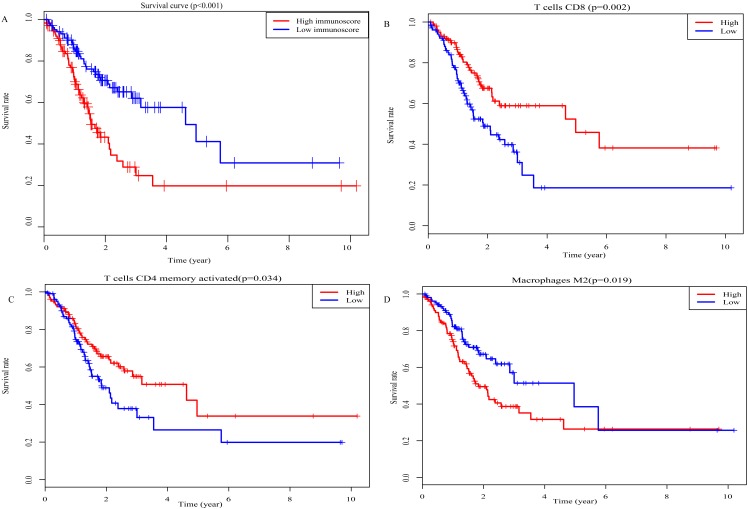
Prognostic values for overall survival. (A) Immunoscore (*P*-values <0.001) (B) CD8 T cells (*P*-values =0.034) (C) Activated memory CD4 T cells (*P*-values =0.002) (D) Macrophages M2 (*P*-values =0.019).

**Figure 7:**
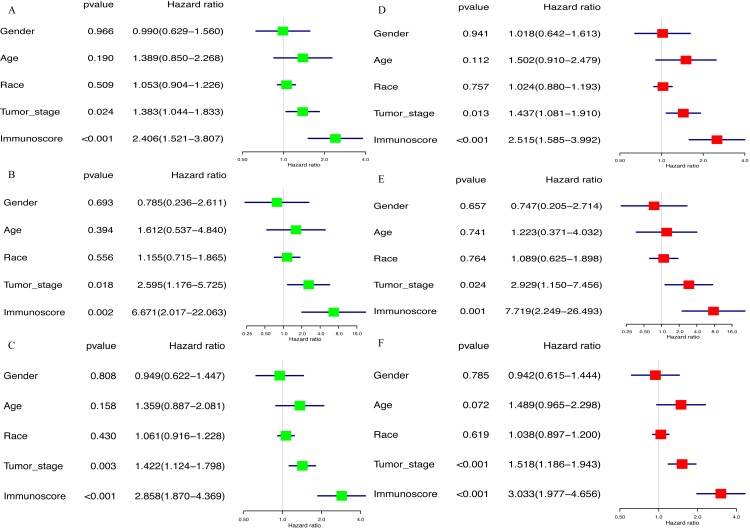
The result of univariable and multivariable Cox regression analysis. (A) The result of univariable Cox regression analysis in TCGA set, (B) ICGC set, (C) whole set. (D) The result of multivariable Cox regression analysis in TCGA set, (E) ICGC set, (F) whole set.

Kaplan–Meier analysis was performed to determine the association between the OS of GC patients and the immunoscore, as well as the proportions of 22 TIICs. The results of survival analysis indicted that the prognosis of GC patients in the high-immunoscore group was significantly worse compared with those in the low-immunoscore group (*P* < 0.001; Fig. 6A). In addition, a low proportion of activated memory CD4 T cells (*P* = 0.002; Fig. 6B) and CD8 T cells (*P* = 0.034; Fig. 6C), along with a high proportion of M2 macrophages (*P* = 0.019; Fig. 6D) was negatively associated with a favorable outcome for GC patients. All results of the univariate Cox regression analysis in TCGA [hazard ratio (HR), 2.406; 95% confidence interval (CI) [1.521–3.807]; *P* < 0.001], the ICGC (HR, 6.671; 95% CI [2.017–22.063]; *P* = 0.002) and whole sets (HR, 2.858; 95% CI [1.870–4.369]; *P* < 0.001) suggested that the association of immunoscore with OS was significant (Figs. 7A–7C). Furthermore, the results of multivariate Cox regression analyses in three sets indicated that the immunoscore was an independent prognostic factor (TCGA set: HR, 2.515; 95% CI [1.585–3.992]; *P* < 0.001; ICGC set: HR, 7.719; 95% CI [2.249–26.493]; *P* = 0.001; whole set: HR, 3.033; 95% CI [1.977–4.656]; *P* < 0.001; Figs. 7D–7F).

### Expression profile of immunomodulators

**Figure 8:**
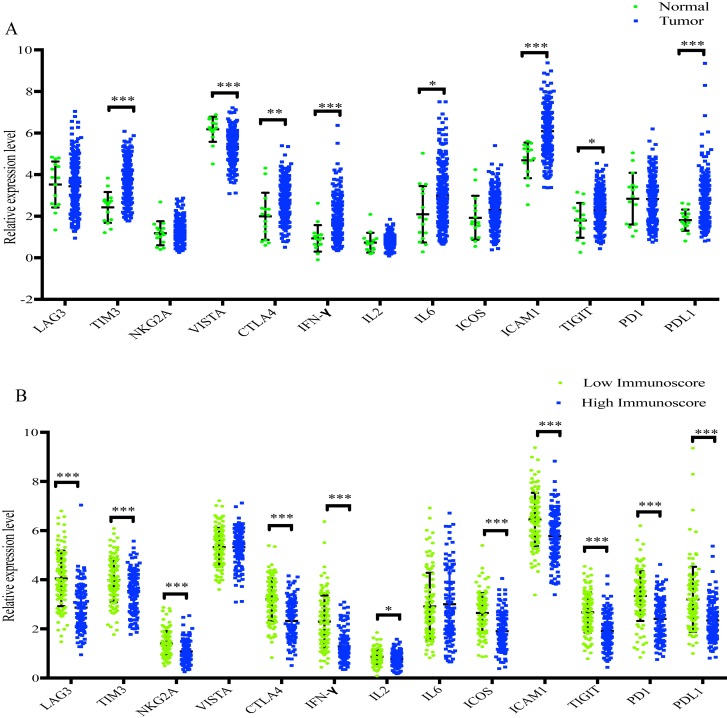
The difference of distribution of immunomodulators. (A) between normal samples and GC samples (B) between low- and high-immunoscore group (*p < 0.05, **p < 0.01, ***p < 0.001).

As presented in Fig. 8A, TIM3, CTLA4, INF-*γ*, IL6, ICAM1, TIGIT and PD-L1 were significantly increased in GC samples compared with normal samples, whereas VISTA was significantly decreased. In addition, when compared with the low-immunoscore group, LAG3, TIM3, NKG2A, CTLA4, INF-*γ*, IL2, ICOS, ICAM1, TIGIT, PD-1 and PD-L1 were all significantly downregulated in the high- immunoscore group (Fig. 8B).

## Discussion

GC, one of the most prevalent digestive malignancies, is the second leading cause of cancer-associated mortality, particularly in developing countries (Sitarz et al., 2018; Chen et al., 2016). The TNM staging system is currently the most relevant clinical index for routine predictions of outcome and treatment. However, emerging studies have reported that the TNM staging system is not completely competent for prognosis stratification (Noh et al., 2014; Zeng et al., 2018). TIICs, as well as the chemokines and cytokines secreted by TIICs, are key regulators of antitumor immune responses (Rohr-Udilova et al., 2018). Previous studies have shown that TIICs, such as lymphocytes, gamma delta T cells, regulatory T cells and macrophages, are significantly associated with the prognosis of GC patients (Zhang et al., 2019; Lee et al., 2018; Yu et al., 2018; Wang et al., 2017; Kang et al., 2017; Jiang et al., 2017; Li et al., 2017; Choi et al., 2016). In addition, Peng et al. (2017) found that NK-cell function in human GC is impaired by monocytes and macrophages in TME and restoring the function of NK cells could prevent GC tumor immune escape. Meanwhile, Koh et al. identified that co-assessment of PD-L1 and CD8+ TILs is clinically relevant, which could provide prognostic significance in stage II/III GCs (Koh et al., 2017). Nevertheless, due to technological limitations of conventional methods, such as IHC and flow cytometry, these previous studies only investigated a few immune cell types and/or a small sample size.

In the present retrospective study, the CIBERSORT method was applied, which is based on deconvolution of bulk gene expression data to synchronously calculate the proportions of 22 TIICs and comprehensively investigate the prognostic impact of 22 TIICs on GC. The present study identified that macrophages (25.3%), resting memory CD4 T cells (16.2%) and CD8 T cells (9.7%) were the highest proportion of immune cells among 22 TIICs in GC. Furthermore, Kaplan–Meier analysis revealed that a low proportion of activated memory CD4 T cells and CD8 T cells predicted a favorable prognosis in GC patients, whereas the opposite result was observed for M2 macrophages. This result is consistent with previous findings that demonstrated the CD8 T cell density in TME is an independent predictor of the OS of GC patients (Wang et al., 2018a; Wang et al., 2018b; Dong et al., 2016) and a high density of M2 macrophages predicts a poor prognosis in GC (Zhang et al., 2012).

To further determine the effect of 22 TIICs on the prognosis of GC patients, LASSO and multivariate Cox regression analysis were used to select the seven best survival-related TIICs (CD8 T cells, activated memory CD4 T cells, gamma delta T cells, monocytes, macrophages M2, neutrophils and eosinophils) and develop an immunoscore formula. The result of survival analysis demonstrated that the high-immunoscore group had a poorer clinical outcome compared with the low-immunoscore group. Furthermore, multivariate Cox regression analysis also demonstrated that variation in immunoscore was highly associated with the clinical outcome of GC patients. A prognostic nomogram for predicting the 3-year OS of GC patients was also built based on immunoscore and clinical information. The calibration plot of the prognostic nomogram demonstrated a good consistency between the prediction and actual observation. In addition, the AUC of the prognostic nomogram was 0.772, which was similar to that in the ICGC set (0.730) and whole set (0.748), and significantly superior to that of TNM staging alone (0.591), indicating that the immunoscore could be used to reinforce the prognostic power of TNM staging. All data suggested that the established prognostic nomogram is suitable for estimating the of 3-year OS of GC patients.

To explore the potential mechanism for the differences in immunoscore, the present study performed an analysis of the DEGs between the low- and high immunoscore groups. A total of 342 DEGs (257 upregulated and 85 downregulated genes) were identified and mainly involved in 13 pathways, such as ‘CAMs’, ‘Chemokine signaling pathway’ and ‘Cytokine-cytokine receptor interaction’. In addition, a MCODE containing 13 genes (CCL20, CXCL10, SAA1, CCL5, CXCR6, CXCL13, CXCL11, CXCL9, ADCY5, PYY, TAS1R3, NMU and CHRM2) was extracted from the PPI network. Functional enrichment analysis demonstrated that four pathways (‘Chemokine signaling pathway’, ‘Cytokine-cytokine receptor interaction’, ‘Toll-like receptor signaling pathway’ and ‘TNF signaling pathway’) were enriched for the13 genes and all of them were highly associated with the human immune system, suggesting that the 13 genes may be the most important targets that cause the variation in immunoscore among GC patients. In addition, CCL20, CXCL10, CCL5, CXCR6, CXCL13, CXCL11 and CXCL9 are chemokines that can recruit immune cells to the tumor microenvironment, which affects tumor immunity and angiogenesis (Lee & Körner, 2019; Tokunaga et al., 2018; Zhang et al., 2018; Singh et al., 2016; Gao et al., 2019).

In recent years, immune checkpoint inhibitors, which function by affecting the immune response, have been approved for the therapy of various cancer types, such as lung cancer, gastric cancer, renal cell carcinoma and hepatocellular carcinoma (Kim & Park, 2019). The present study identified 11 immunomodulators (LAG3, TIM3, NKG2A, CTLA4, INF-*γ*, IL2, ICOS, ICAM1, TIGIT, PD-1 and PD-L1) that were significantly decreased in the high-immunoscore group compared with the low-immunoscore group, which may assist with the development of effective therapeutics.

Although the prognostic nomogram demonstrated a good predictive accuracy for GC patients in the present study, there are a few limitations to be addressed. First, as all cases were obtained from public databases, it was not possible to collect all information of the patients, such as history of treatment with anti-inflammatory drugs. Second, the potential of selection bias could not be excluded. Third, all gene expression profiles used were derived from core regions of cancer samples, which means deviation in immune infiltration cells between the core and invasive margin of tumors could not be analyzed. Finally, no experimental studies were conducted to confirm the findings of the present study. Therefore, further investigations both *in vitro* and *in vivo* are required to support the present results.

## Conclusions

In summary, the present study comprehensively analyzed gene expression profiles of GC from TCGA database to quantify the proportions of 22 TIICs with the CIBERSORT algorithm. The seven best survival-related TIICs were screened out to develop an immunoscore formula. By combining the immunoscore and clinical information, a prognostic nomogram was built for predicting the 3-year OS. The results suggested the immunoscore could be used to reinforce the clinical outcome prediction ability of the TNM staging system and provide a convenient tool for risk assessment and treatment selection for GC patients. However, further experimental research both *in vitro* and *in vivo* is required to examine the present findings.

##  Supplemental Information

10.7717/peerj.7993/supp-1 Table S1 Gene Ontology analysis of 342* DEGs*

10.7717/peerj.7993/supp-2 Figure S1 Volcano plot of 342 DEGs with |*logFC*| > 2 and *P*-value <0.01 Red dots represent up-regulated DEGs and green dots represent down-regulated DEGs.

10.7717/peerj.7993/supp-3 Figure S2 The pathways enriched by 13 genes in MCODE

## Abbreviations

GC: Gastric cancer
TIICs: tumor-infiltrating immune cells
AUC: the area under the curve
ROC: the receiver operating characteristic curve
AJCC/UICC: American Joint Committee on Cancer and International Union against Cancer
TME: The tumor microenvironment
IHC: Immunohistochemistry
TCGA: The Cancer Genome Altas
ICGC: international cancer genomics consortium
DEGs: differentially expressed genes
KEGG: Kyoto Encyclopedia of Genes and Genomes
GO: gene ontology
PPI: Protein-protein Interaction
MCODE: Molecular Complex Detection
CI: Confidence interval
HR: Hazard ratio

## Additional Information and Declarations

### Data Availability

The following information was supplied regarding data availability: The data is available at the Cancer Genome Altas (TCGA, https://cancergenome.nih.gov) under TCGA search term: TCGA-STAD and from the International cancer genomics consortium (ICGC, https://icgc.org) using ICGC search term: GACA-JP.

## References

[ref-1] Bense RD, Sotiriou C, Piccart-Gebhart MJ, Haanen JBAG, Van Vugt MATM, De Vries EGE, Schröder CP, Fehrmann RSN (2016). Relevance of tumor-infiltrating immune cell composition and functionality for disease outcome in breast cancer. Journal of the National Cancer Institute.

[ref-2] Chen J, Zhang J, Li X, Zhang C, Zhang H, Jin J, Dai D (2016). Downregulation of ADAMTS8 by DNA hypermethylation in gastric cancer and its clinical significance. BioMed Research International.

[ref-3] Choi HS, Ha SY, Kim HM, Ahn SM, Kang MS, Kim KM, Choi MG, Lee JH, Sohn TS, Bae JM, Kim S, Kang ES (2016). The prognostic effects of tumor infiltrating regulatory T cells and myeloid derived suppressor cells assessed by multicolor flow cytometry in gastric cancer patients. Oncotarget.

[ref-4] Dong J, Li J, Liu S, Feng X, Chen S, Zhou Z, Chen Y, Zhang X (2016). Prognostic potential of an immune score based on the density of CD8+ T cells, CD20+ B cells, and CD33+/p-STAT1+ double-positive cells and HMGB1 expression within cancer nests in stage IIIA gastric cancer patients. Chinese Journal of Cancer Research.

[ref-5] Gao Q, Wang S, Chen X, Cheng S, Zhang Z, Li F, Huang L, Yang Y, Zhou B, Yue D, Wang D, Cao L, Maimela NR, Zhang B, Yu J, Wang L, Zhang Y (2019). Cancer-cell-secreted CXCL11 promoted CD8+ T cells infiltration through docetaxel-induced-release of HMGB1 in NSCLC. Journal for ImmunoTherapy of Cancer.

[ref-6] Jiang W, Liu K, Guo Q, Cheng J, Shen L, Cao Y, Wu J, Shi J, Cao H, Liu B, Tao K, Wang G, Cai K (2017). Tumor-infiltrating immune cells and prognosis in gastric cancer: a systematic review and meta-analysis. Oncotarget.

[ref-7] Jiang Y, Zhang Q, Hu Y, Li T, Yu J, Zhao L, Ye G, Deng H, Mou T, Cai S, Zhou Z, Liu H, Chen G, Li G, Qi X (2018). ImmunoScore signature a prognostic and predictive tool in gastric cancer. Annals of Surgery.

[ref-8] Kang BW, Kim JG, Lee IH, Bae HI, Seo AN (2017). Clinical significance of tumor-infiltrating lymphocytes for gastric cancer in the era of immunology. World Journal of Gastrointestinal Oncology.

[ref-9] Kim HD, Park SH (2019). Immunological and clinical implications of immune checkpoint blockade in human cancer. Archives of Pharmacal Research.

[ref-10] Koh J, Ock CY, Kim JW, Nam SK, Kwak Y, Yun S, Ahn SH, Park DJ, Kim HH, Kim WH, Lee HS (2017). Clinicopathologic implications of immune classification by PD-L1 expression and CD8-positive tumor-infiltrating lymphocytes in stage II and III gastric cancer patients. Oncotarget.

[ref-11] Lee AYS, Körner H (2019). The CCR6-CCL20 axis in humoral immunity and T-B cell immunobiology. Immunobiology.

[ref-12] Lee JS, Won HS, Sun S, Hong JH, Ko YH (2018). Prognostic role of tumor-infiltrating lymphocytes in gastric cancer: a systematic review and meta-analysis. Medicine.

[ref-13] Li JQ, Yu XJ, Wang YC, Huang LY, Liu CQ, Zheng L, Fang YJ, Xu J (2017). Distinct patterns and prognostic values of tumor-infiltrating macrophages in hepatocellular carcinoma and gastric cancer. Journal of Translational Medicine.

[ref-14] Liu X, Wu S, Yang Y, Zhao M, Zhu G, Hou Z (2017). The prognostic landscape of tumor-infiltrating immune cell and immunomodulators in lung cancer. Biomedicine and Pharmacotherapy.

[ref-15] Liu Z, Zhu Y, Xu L, Zhang J, Xie H, Fu H, Zhou Q, Chang Y, Dai B, Xu J (2018). Tumor stroma-infiltrating mast cells predict prognosis and adjuvant chemotherapeutic benefits in patients with muscle invasive bladder cancer. Oncoimmunology.

[ref-16] Newman AM, Liu CL, Green MR, Gentles AJ, Feng W, Xu Y, Hoang CD, Diehn M, Alizadeh AA (2015). Robust enumeration of cell subsets from tissue expression profiles. Nature Methods.

[ref-17] Noh SH, Park SR, Yang HK, Chung HC, Chung IJ, Kim SW, Kim HH, Choi JH, Kim HK, Yu W, Lee JI, Shin DB, Ji J, Chen JS, Lim Y, Ha S, Bang YJ (2014). CLASSIC trial investigators adjuvant capecitabine plus oxaliplatin for gastric cancer after D2 gastrectomy (CLASSIC): 5-year follow-up of an open- label, randomised phase 3 trial. The Lancet Oncology.

[ref-18] Park JY, Forman D, Waskito LA, Yamaoka Y, Crabtree JE (2018). Epidemiology of helicobacter pylori and CagA-positive infections and global variations in gastric cancer. Toxins.

[ref-19] Peng LS, Zhang JY, Teng YS, Zhao YL, Wang TT, Mao FY, Lv YP, Cheng P, Li WH, Chen N, Duan M, Chen W, Guo G, Zou QM, Zhuang Y (2017). Tumor-associated monocytes/macrophages impair NK-cell function via TGF*β*1 in human gastric cancer. Cancer Immunology Research.

[ref-20] R Core Team (2018). https://www.R-project.org/.

[ref-21] Rohr-Udilova N, Klinglmüller F, Schulte-Hermann R, Stift J, Herac M, Salzmann M, Finotello F, Timelthaler G, Oberhuber G, Pinter M, Reiberger T, Jensen-Jarolim E, Eferl R, Trauner M (2018). Deviations of the immune cell landscape between healthy liver and hepatocellular carcinoma. Scientific Reports.

[ref-22] Singh R, Kapur N, Mir H, Singh N, Lillard Jr JW, Singh S (2016). CXCR6-CXCL16 axis promotes prostate cancer by mediating cytoskeleton rearrangement via Ezrin activation and *α*v *β*3 integrin clustering. Oncotarget.

[ref-23] Sitarz R, Skierucha M, Mielko J, Offerhaus GJA, Maciejewski R, Polkowski WP (2018). Gastric cancer: epidemiology, prevention, classification, and treatment. Cancer Management and Research.

[ref-24] Szklarczyk D, Gable AL, Lyon D, Junge A, Wyder S, Huerta-Cepas J, Simonovic M, Doncheva NT, Morris JH, Bork P, Jensen LJ, Mering CV (2019). STRING v11: protein-protein association networks with increased coverage, supporting functional discovery in genome-wide experimental dataset. Nucleic Acids Research.

[ref-25] Tokunaga R, Zhang W, Naseem M, Puccini A, Berger MD, Soni S, McSkane M, Baba H, Lenz HJ (2018). CXCL9, CXCL10, CXCL11/CXCR3 axis for immune activation—a target for novel cancer therapy. Cancer Treatment Reviews.

[ref-26] Wang J, Lin C, Li H, Li R, Wu Y, Liu H, Zhang H, He H, Zhang W, Xu J (2017). Tumor-infiltrating *γδ*T cells predict prognosis and adjuvant chemotherapeutic benefit in patients with gastric cancer. Oncoimmunology.

[ref-27] Wang L, Chang Y, Xu J, Zhang Q (2016). Predictive significance of serum level of vascular endothelial growth factor in gastric cancer patients. BioMed Research International.

[ref-28] Wang Y, Xiao S, Wang B, Li Y, Chen Q (2018a). Knockdown of lncRNA TP73-AS1 inhibits gastric cancer cell proliferation and invasion via the WNT/*β*-catenin signaling pathway. Oncology Letters.

[ref-29] Wang Y, Zhu C, Song W, Li J, Zhao G, Cao H (2018b). PD-L1 expression and CD8+ T cell infiltration predict a favorable prognosis in advanced gastric cancer. Journal of Immunology Research.

[ref-30] Wei J, Wang Z, Wang Z, Yang Y, Fu C, Zhu J, Jiang D (2017). MicroRNA-31 function as a suppressor was regulated by epigenetic mechanisms in gastric cancer. BioMed Research International.

[ref-31] Wu F, Gao H, Liu K, Gao B, Ren H, Li Z, Liu F (2019). The lncrna ZeB2-as1 is upregulated in gastric cancer and affects cell proliferation and invasion via mir-143-5p/hiF-1*α* axis. OncoTargets and Therapy.

[ref-32] Xiong Y, Wang K, Zhou H, Peng L, You W, Fu Z (2018). Profiles of immune infiltration in colorectal cancer and their clinical significant: a gene expression-based study. Cancer Medicine.

[ref-33] Yang N, Zhu S, Lv X, Qiao Y, Liu YJ, Chen J (2018). MicroRNAs: pleiotropic regulators in the tumor microenvironment. Frontiers in Immunology.

[ref-34] Yu PC, Long D, Liao CC, Zhang S (2018). Association between density of tumor-infiltrating lymphocytes and prognoses of patients with gastric cancer. Medicine.

[ref-35] Zeng D, Zhou R, Yu Y, Luo Y, Zhang J, Sun H, Zeng D, Zhou R, Yu Y, Luo Y, Zhang J, Sun H, Bin J, Liao Y, Rao J, Zhang Y, Liao W (2018). Gene expression profiles for a prognostic immunoscore in gastric cancer. British Journal of Surgery.

[ref-36] Zhang D, He W, Wu C, Tan Y, He Y, Xu B, Chen L, Li Q, Jiang J (2019). Scoring system for tumor-infiltrating lymphocytes and its prognostic value for gastric cancer. Frontiers in Immunology.

[ref-37] Zhang QW, Liu L, Gong CY, Shi HS, Zeng YH, Wang XZ, Zhao YW, Wei YQ (2012). Prognostic significance of tumor-associated macrophages in solid tumor: a meta-analysis of the literature. PLOS ONE.

[ref-38] Zhang S, Zhong M, Wang C, Xu Y, Gao WQ, Zhang Y (2018). CCL5-deficiency enhances intratumoral infiltration of CD8+ T cells in colorectal cancer. Cell Death & Disease.

[ref-39] Zhou R, Zhang J, Zeng D, Sun H, Rong X, Shi M, Bin J, Liao Y, Liao W (2019). Immune cell infiltration as a biomarker for the diagnosis and prognosis of stage I–III colon cancer. Cancer Immunology and Immunotherapy.

