# Supplementary material for: The landscape and prognostic value of tumor-infiltrating immune cells in gastric cancer
Source: PeerJ. 2019 Dec 10;7:e7993. doi: 10.7717/peerj.7993 (PMC6910118; doi:10.7717/peerj.7993)
Supplement: Table S1 [file peerj-07-7993-s001.docx]

Table S1. Gene Ontology analysis of 342 *DEGs*

| GO | Terms | GeneRatio | Count | p-value |
| --- | --- | --- | --- | --- |
| BP | muscle contraction | 31/279 | 31 | <0.001 |
| BP | muscle system process | 34/279 | 34 | <0.001 |
| BP | blood circulation | 28/279 | 28 | <0.001 |
| BP | circulatory system process | 28/279 | 28 | <0.001 |
| BP | antimicrobial humoral response | 14/279 | 14 | <0.001 |
| BP | regulation of heart contraction | 18/279 | 18 | <0.001 |
| BP | antimicrobial humoral immune response mediated by antimicrobial peptide | 11/279 | 11 | <0.001 |
| BP | regulation of blood circulation | 19/279 | 19 | <0.001 |
| BP | heart contraction | 18/279 | 18 | <0.001 |
| BP | muscle organ development | 23/279 | 23 | <0.001 |
| BP | heart process | 18/279 | 18 | <0.001 |
| BP | regulation of metal ion transport | 22/279 | 22 | <0.001 |
| BP | regulation of muscle contraction | 14/279 | 14 | <0.001 |
| BP | regulation of muscle system process | 17/279 | 17 | <0.001 |
| BP | regulation of cation transmembrane transport | 19/279 | 19 | <0.001 |
| BP | potassium ion transport | 16/279 | 16 | <0.001 |
| BP | chemokine-mediated signaling pathway | 10/279 | 10 | <0.001 |
| BP | cellular potassium ion transport | 15/279 | 15 | <0.001 |
| BP | potassium ion transmembrane transport | 15/279 | 15 | <0.001 |
| BP | positive regulation of transmembrane transport | 14/279 | 14 | <0.001 |
| BP | regulation of ion transmembrane transport | 22/279 | 22 | <0.001 |
| BP | response to chemokine | 10/279 | 10 | <0.001 |
| BP | cellular response to chemokine | 10/279 | 10 | <0.001 |
| BP | positive regulation of ion transport | 16/279 | 16 | <0.001 |
| BP | regulation of heart rate | 10/279 | 10 | <0.001 |
| BP | T cell chemotaxis | 6/279 | 6 | <0.001 |
| BP | T cell migration | 8/279 | 8 | <0.001 |
| BP | muscle cell differentiation | 19/279 | 19 | <0.001 |
| BP | lymphocyte chemotaxis | 8/279 | 8 | <0.001 |
| BP | humoral immune response | 18/279 | 18 | <0.001 |
| BP | positive regulation of cation transmembrane transport | 11/279 | 11 | <0.001 |
| BP | actin-mediated cell contraction | 10/279 | 10 | <0.001 |
| BP | regulation of protein secretion | 20/279 | 20 | 0.001 |
| BP | second-messenger-mediated signaling | 20/279 | 20 | 0.001 |
| BP | granulocyte chemotaxis | 10/279 | 10 | 0.001 |
| BP | multicellular organismal signaling | 12/279 | 12 | 0.001 |
| BP | positive regulation of ion transmembrane transport | 11/279 | 11 | 0.001 |
| BP | regulation of calcium ion transport | 14/279 | 14 | 0.001 |
| BP | regulation of calcium ion transport into cytosol | 9/279 | 9 | 0.002 |
| BP | striated muscle cell differentiation | 15/279 | 15 | 0.002 |
| BP | neutrophil chemotaxis | 9/279 | 9 | 0.002 |
| BP | regulation of cytosolic calcium ion concentration | 17/279 | 17 | 0.002 |
| BP | regulation of release of sequestered calcium ion into cytosol | 8/279 | 8 | 0.002 |
| BP | regulation of T cell migration | 6/279 | 6 | 0.002 |
| BP | regulation of peptide secretion | 20/279 | 20 | 0.002 |
| BP | smooth muscle contraction | 9/279 | 9 | 0.002 |
| BP | regulation of potassium ion transport | 9/279 | 9 | 0.002 |
| BP | lymphocyte migration | 9/279 | 9 | 0.002 |
| BP | calcium-mediated signaling | 13/279 | 13 | 0.002 |
| BP | cellular potassium ion homeostasis | 4/279 | 4 | 0.002 |
| BP | granulocyte migration | 10/279 | 10 | 0.002 |
| BP | digestion | 10/279 | 10 | 0.002 |
| BP | actin filament-based movement | 10/279 | 10 | 0.003 |
| BP | positive regulation of T cell apoptotic process | 4/279 | 4 | 0.003 |
| BP | cardiac conduction | 9/279 | 9 | 0.003 |
| BP | monocyte chemotaxis | 7/279 | 7 | 0.003 |
| BP | neutrophil migration | 9/279 | 9 | 0.003 |
| BP | striated muscle contraction | 11/279 | 11 | 0.003 |
| BP | cellular calcium ion homeostasis | 19/279 | 19 | 0.003 |
| BP | potassium ion homeostasis | 5/279 | 5 | 0.003 |
| BP | calcium ion transport | 18/279 | 18 | 0.003 |
| BP | membrane depolarization | 8/279 | 8 | 0.003 |
| BP | regulation of potassium ion transmembrane transport | 8/279 | 8 | 0.003 |
| BP | cardiac muscle cell contraction | 7/279 | 7 | 0.003 |
| BP | release of sequestered calcium ion into cytosol | 9/279 | 9 | 0.003 |
| BP | negative regulation of transport | 20/279 | 20 | 0.003 |
| BP | positive regulation of cytosolic calcium ion concentration | 15/279 | 15 | 0.004 |
| BP | negative regulation of sequestering of calcium ion | 9/279 | 9 | 0.004 |
| BP | positive regulation of T cell migration | 5/279 | 5 | 0.004 |
| BP | calcium ion homeostasis | 19/279 | 19 | 0.004 |
| BP | regulation of T cell chemotaxis | 4/279 | 4 | 0.004 |
| BP | divalent inorganic cation transport | 19/279 | 19 | 0.004 |
| BP | regulation of membrane potential | 18/279 | 18 | 0.004 |
| BP | regulation of sequestering of calcium ion | 9/279 | 9 | 0.004 |
| BP | calcium ion transport into cytosol | 10/279 | 10 | 0.004 |
| BP | peptide hormone secretion | 13/279 | 13 | 0.004 |
| BP | T cell apoptotic process | 6/279 | 6 | 0.004 |
| BP | regulation of tolerance induction | 4/279 | 4 | 0.004 |
| BP | cellular sodium ion homeostasis | 4/279 | 4 | 0.004 |
| BP | sequestering of calcium ion | 9/279 | 9 | 0.004 |
| BP | cell junction organization | 14/279 | 14 | 0.005 |
| BP | cardiac muscle cell action potential | 7/279 | 7 | 0.005 |
| BP | positive regulation of lymphocyte apoptotic process | 4/279 | 4 | 0.005 |
| BP | adenylate cyclase-modulating G-protein coupled receptor signaling pathway | 12/279 | 12 | 0.005 |
| BP | cellular divalent inorganic cation homeostasis | 19/279 | 19 | 0.006 |
| BP | export across plasma membrane | 5/279 | 5 | 0.006 |
| BP | release of sequestered calcium ion into cytosol by endoplasmic reticulum | 5/279 | 5 | 0.006 |
| BP | cytosolic calcium ion transport | 10/279 | 10 | 0.006 |
| BP | regulation of hormone secretion | 13/279 | 13 | 0.006 |
| BP | positive regulation of response to external stimulus | 14/279 | 14 | 0.006 |
| BP | calcium ion transmembrane import into cytosol | 9/279 | 9 | 0.006 |
| BP | positive regulation of secretion by cell | 16/279 | 16 | 0.007 |
| BP | positive regulation of lymphocyte migration | 5/279 | 5 | 0.007 |
| BP | response to interferon-gamma | 11/279 | 11 | 0.008 |
| BP | epidermis development | 18/279 | 18 | 0.008 |
| BP | divalent metal ion transport | 18/279 | 18 | 0.008 |
| BP | myeloid leukocyte migration | 11/279 | 11 | 0.008 |
| BP | regulation of T cell activation | 14/279 | 14 | 0.008 |
| BP | monovalent inorganic cation homeostasis | 9/279 | 9 | 0.008 |
| BP | hormone secretion | 14/279 | 14 | 0.008 |
| BP | eosinophil chemotaxis | 4/279 | 4 | 0.008 |
| BP | regulation of lymphocyte migration | 6/279 | 6 | 0.008 |
| BP | cell junction assembly | 12/279 | 12 | 0.009 |
| BP | regulation of myotube differentiation | 6/279 | 6 | 0.009 |
| BP | cytolysis | 5/279 | 5 | 0.009 |
| BP | cornification | 8/279 | 8 | 0.009 |
| BP | mononuclear cell migration | 7/279 | 7 | 0.009 |
| BP | regulation of cardiac muscle contraction by regulation of the release of sequestered calcium ion | 4/279 | 4 | 0.009 |
| BP | regulation of lymphocyte activation | 18/279 | 18 | 0.009 |
| BP | potassium ion import across plasma membrane | 5/279 | 5 | 0.010 |
| BP | hormone transport | 14/279 | 14 | 0.010 |
| BP | regulation of smooth muscle contraction | 6/279 | 6 | 0.010 |
| CC | contractile fiber part | 26/298 | 26 | <0.001 |
| CC | contractile fiber | 26/298 | 26 | <0.001 |
| CC | myofibril | 25/298 | 25 | <0.001 |
| CC | sarcomere | 23/298 | 23 | <0.001 |
| CC | Z disc | 18/298 | 18 | <0.001 |
| CC | I band | 18/298 | 18 | <0.001 |
| CC | sarcolemma | 16/298 | 16 | <0.001 |
| CC | costamere | 7/298 | 7 | <0.001 |
| CC | cell-substrate junction | 18/298 | 18 | 0.002 |
| CC | cell-substrate adherens junction | 17/298 | 17 | 0.005 |
| CC | membrane raft | 14/298 | 14 | 0.006 |
| CC | membrane microdomain | 14/298 | 14 | 0.006 |
| CC | anchored component of membrane | 10/298 | 10 | 0.006 |
| CC | membrane region | 14/298 | 14 | 0.008 |
| CC | external side of plasma membrane | 15/298 | 15 | 0.008 |
| CC | actin cytoskeleton | 18/298 | 18 | 0.008 |
| CC | cornified envelope | 6/298 | 6 | 0.008 |
| CC | focal adhesion | 16/298 | 16 | 0.008 |
| CC | transmembrane transporter complex | 14/298 | 14 | 0.008 |
| CC | cation channel complex | 11/298 | 11 | 0.008 |
| CC | transporter complex | 14/298 | 14 | 0.009 |
| MF | receptor ligand activity | 30/275 | 30 | <0.001 |
| MF | G-protein coupled receptor binding | 20/275 | 20 | <0.001 |
| MF | chemokine activity | 9/275 | 9 | <0.001 |
| MF | cytokine activity | 17/275 | 17 | <0.001 |
| MF | chemokine receptor binding | 9/275 | 9 | <0.001 |
| MF | structural constituent of muscle | 7/275 | 7 | <0.001 |
| MF | CXCR chemokine receptor binding | 4/275 | 4 | 0.001 |
| MF | actin binding | 19/275 | 19 | 0.002 |
| MF | cytokine receptor binding | 15/275 | 15 | 0.002 |
| MF | potassium ion transmembrane transporter activity | 11/275 | 11 | 0.002 |
| MF | CCR chemokine receptor binding | 6/275 | 6 | 0.002 |
| MF | potassium channel regulator activity | 6/275 | 6 | 0.006 |
| MF | enzyme inhibitor activity | 16/275 | 16 | 0.009 |
| MF | ion channel regulator activity | 8/275 | 8 | 0.009 |

DEGs; differentially expressed genes, BP; Biological Process, CC; Cellular Component, MF; Molecular Function
