# Supplementary figures and images for: The landscape and prognostic value of tumor-infiltrating immune cells in gastric cancer
Source: PeerJ. 2019 Dec 10;7:e7993. doi: 10.7717/peerj.7993 (PMC6910118; doi:10.7717/peerj.7993)

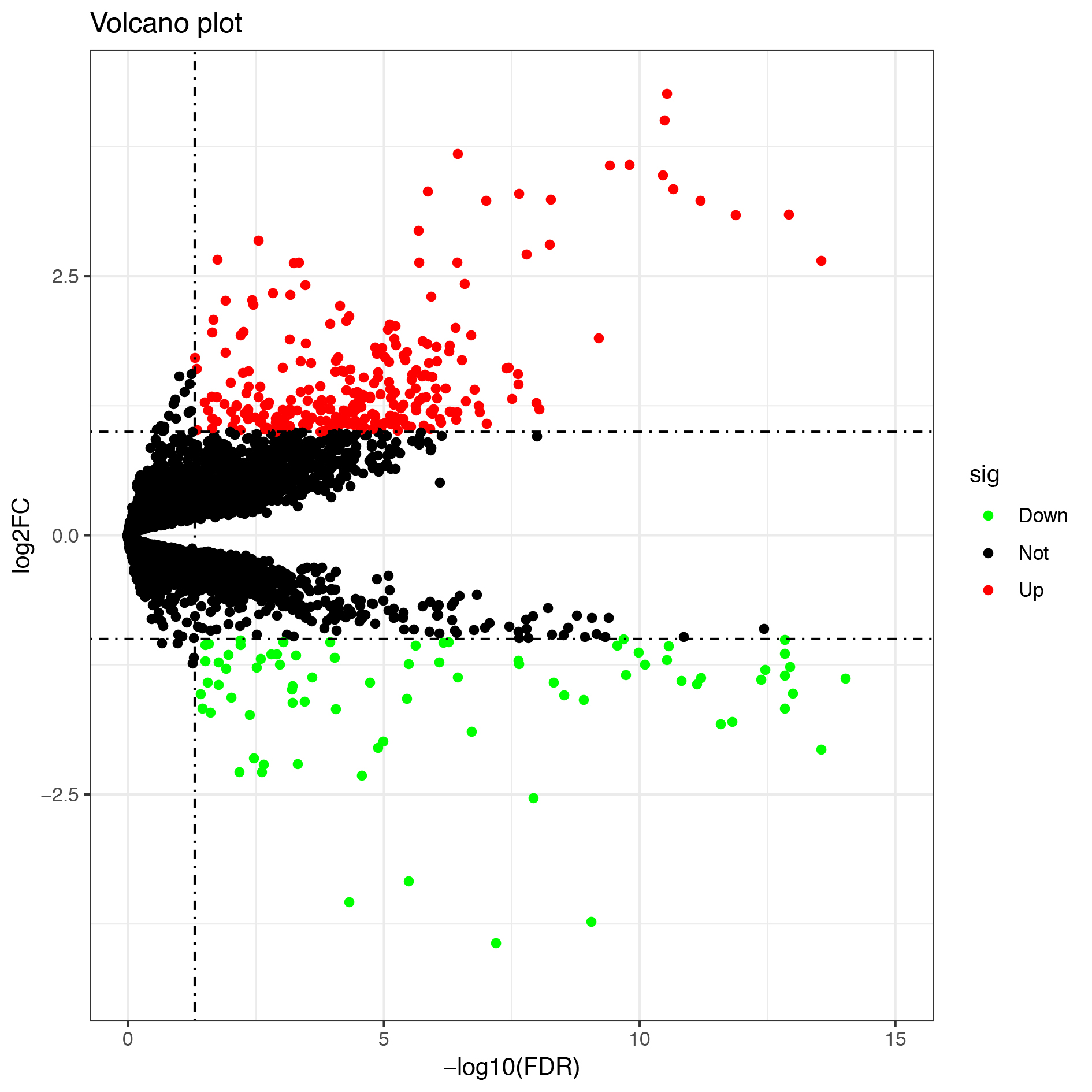

Supplement: Figure S1 — Red dots represent up-regulated DEGs and green dots represent down-regulated DEGs. [file peerj-07-7993-s002.jpg]

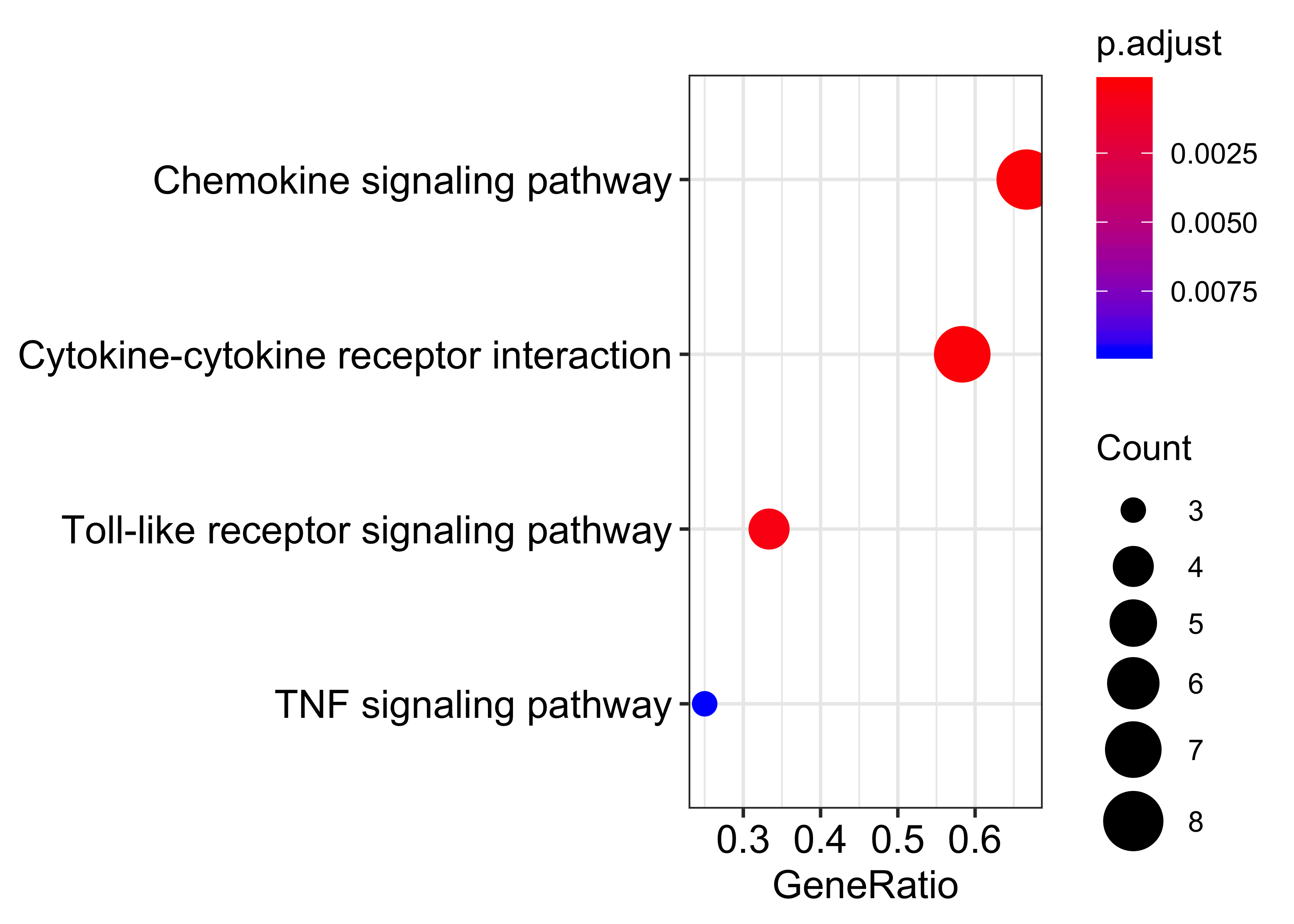

Supplement: Figure S2 [file peerj-07-7993-s003.jpg]
